# Palmitoylethanolamide induces microglia changes associated with increased migration and phagocytic activity: involvement of the CB2 receptor

**DOI:** 10.1038/s41598-017-00342-1

**Published:** 2017-03-23

**Authors:** F. Guida, L. Luongo, S. Boccella, M. E. Giordano, R. Romano, G. Bellini, I. Manzo, A. Furiano, A. Rizzo, R. Imperatore, F. A. Iannotti, E. D’Aniello, F. Piscitelli, F. sca Rossi, L. Cristino, V. Di Marzo, V. de Novellis, S. Maione

**Affiliations:** 1 Department of Experimental Medicine, Section of Pharmacology L. Donatelli, Università degli Studi della Campania “Luigi Vanvitelli” (Ex SUN), 80138 Naples, Italy; 2 Department of Women, Child and General and Specialistic Surgery, Università degli Studi della Campania “Luigi Vanvitelli” (Ex SUN), 80138 Naples, Italy; 3 Department of Experimental Medicine, Section of Microbiology and Clinical Microbiology, Università degli Studi della Campania “Luigi Vanvitelli” (Ex SUN), 80138 Naples, Italy; 4 Institute of Biomolecular Chemistry, Consiglio Nazionale delle Ricerche, Pozzuoli, Italy; 5 Endocannabinoid Research Group, Institute of Biomolecular Chemistry, C.N.R., Pozzuoli, Italy; 6 Department of Science and Technology, University of Sannio, Benevento, Italy

## Abstract

The endogenous fatty acid amide palmitoylethanolamide (PEA) has been shown to exert anti-inflammatory actions mainly through inhibition of the release of pro-inflammatory molecules from mast cells, monocytes and macrophages. Indirect activation of the endocannabinoid (eCB) system is among the several mechanisms of action that have been proposed to underlie the different effects of PEA *in vivo*. In this study, we used cultured rat microglia and human macrophages to evaluate whether PEA affects eCB signaling. PEA was found to increase CB2 mRNA and protein expression through peroxisome proliferator-activated receptor-α (PPAR-α) activation. This novel gene regulation mechanism was demonstrated through: (i) pharmacological PPAR-α manipulation, (ii) PPAR-α mRNA silencing, (iii) chromatin immunoprecipitation. Moreover, exposure to PEA induced morphological changes associated with a reactive microglial phenotype, including increased phagocytosis and migratory activity. Our findings suggest indirect regulation of microglial CB2R expression as a new possible mechanism underlying the effects of PEA. PEA can be explored as a useful tool for preventing/treating the symptoms associated with neuroinflammation in CNS disorders.

## Introduction

Microglial cells are the resident immune cells responsible for maintaining homeostasis in the Central Nervous System (CNS). The activation of microglia is part of an early defense mechanism following injury or disease^1^. In pathological conditions, these cells assume an activated state characterized by morphological rearrangement, proliferation, chemotaxis towards the site of damage, and release of mediators^2^. Increasing evidence indicates that microglial activation is heterogeneous. In fact, depending on the phenotype assumed, microglia can elicit either cytotoxic or neuroprotective effects^3^. In addition to the inflammatory component, microglia are also considered to be the professional phagocytes in the CNS, responsible for removing dying or apoptotic cells, myelin debris, and bacteria^4^. The majority of pathogens that reach the brain are physiologically eliminated or sequestered in a latent form by microglia. However, highly virulent strains can produce a progressive inflammatory response associated with increased expression of cytokines and dysregulation of microglia cells, which is an hallmark of early stage neurodegeneration^5^.

It is well known that microglia express components of the endocannabinoid (eCB) system, including receptors, ligands, and metabolic enzymes. In particular, the cannabinoid (CB) type 2 receptor (CB2R) is tightly regulated on microglial surfaces during several pathological states^6, 7^. Indeed, selective CB2R stimulation inhibits microglial reactivity and promotes a neuroprotective phenotype^8, 9^.

Palmitoylethanolamide (PEA) is an eCB-like compound, which belongs to the class of long chain fatty acid ethanolamides that have been shown to have cytoprotective and anti-inflammatory activity. This is achieved by inhibition of the release of pro-inflammatory molecules from mast cells, monocytes, and macrophages^10, 11^. PEA is produced by neurons and glial cells in the CNS and is involved in the endogenous neuroprotective mechanisms that are activated following tissue damage or inflammation^12–14^. PEA exerts anti-inflammatory and analgesic effects through several molecular and cellular mechanisms^15–17^. Although it does not bind to CB receptors with high affinity^18^, selective blockers of CB1 or CB2 receptors have been shown to inhibit a multitude of its effects *in vivo* ^19, 20^. In fact, PEA has been shown to act on several targets, including GPR55, transient receptor potential vannilloid type-1 (TRPV1) channels, fatty acid amide hydrolase (FAAH), and, last but not least, peroxisome proliferator-activated receptor-α (PPAR-α)^21, 22^. However, the mechanism by which PEA acts on these receptors is highly debated. The apparent multitarget action of PEA inspired us to investigate the possible mechanisms underlying the role of CBRs activation in response to PEA-mediated effects.

In the present study, we aimed to determine if PEA affects eCB signaling, by focusing on the expression of CB2R in microglia cells, because of the well established role of this receptor in immune cells^6^. Using pharmacological, biochemical, and computational approaches, we investigated the involvement of PPAR-α in CB2R regulation in response to PEA. Additionally, the capability of PEA to regulate the ability of microglia to migrate and phagocytose as a possible consequence of CB2R activation was also evaluated. The subsequent discovery of a further mechanism of action for PEA paves the way to investigate its possible implication in several chronic neuroinflammatory diseases.

## Results

### PEA enhances CB2R expression via PPAR-α activation

Control experiments verified that PEA treatment (1–100 nM for 24 hours) did not induce significant disruption of cellular integrity, as measured by release of the cytoplasmic enzyme lactate dehydrogenase (LDH) (not shown).

**Figure 1:**
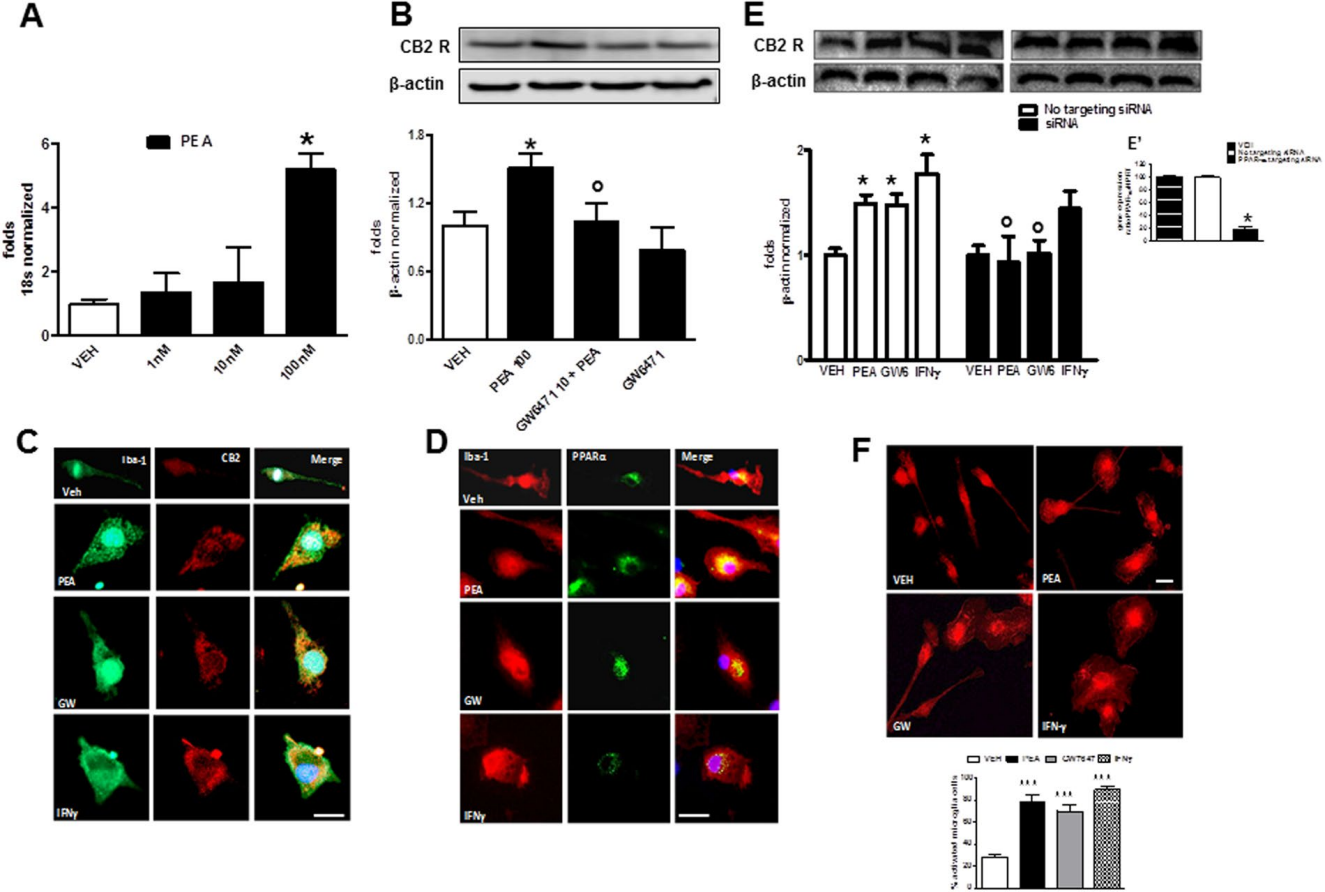
PEA induces CB2R expression via PPAR-α activation. (**A**) Real-time PCR analyses of CB2m-RNA in cultured microglia following incubation with PEA (1, 10 and 100 nM for 24 hours), using 18S as a loading control. Data are shown as mean ± SD (n = 6–8). *P < 0.05 compared to vehicle-treated cells. One way ANOVA, post-hoc Tukey’s. (**B**) Representative western blot image and related quantification showing the expression of CB2R in microglial cell lysates following incubation with vehicle, PEA (100 nM), GW6471 (10 µM) + PEA, or GW6471 alone, using β-actin as a loading control. Data are shown as mean ± SD (n = 6–8). *P < 0.05 compared to vehicle-treated cells, °P < 0.05 compared to PEA-treated cells. One way ANOVA, post-hoc Tukey’s. **(C**) CB2R expression (red) in Iba-1 labeled microglia (green) following incubation with vehicle, PEA (100 nM), GW7647 (1 µM), IFNγ (300 U/ml). Scale bar = 25 μm. (**D**) PPAR-α expression (green) in microglia cells (red) following incubation with vehicle, PEA (100 nM), GW7647 (1 µM), IFNγ (300 U/ml). Scale bar = 25 μm. (**E**) Representative western blot image and related quantification showing the expression of CB2R in microglial cell lysates incubated for 24 hours with vehicle, PEA (100 nM), GW7647 (1 µM), IFNγ (300 U/ml), in presence of siRNA or non-targeting siRNA. Data are shown as mean ± SD (n = 6). *P < 0.05 compared to vehicle, °P < 0.05 compared to relative control within non-targeting siRNA group. One way ANOVA, post-hoc Tukey’s. (**E’**) Real-time PCR analyses of PPAR-α/HPRT ratio in PPAR-α silencing (siRNA) or non-targeting siRNA conditions, compared with vehicle. Data are shown as means ± SD (n = 6). *P < 0.05, One way ANOVA, post-hoc Tukey’s. **(F)** Representative staining and related quantification of microglia cells in Iba-1 labeled microglia incubated for 24 hours with PEA (100 nM), GW7647 (1 µM) or IFNγ (300 U/ml), compared with vehicle-treated cells. Morphological evaluations measured as a percentage of activated cells over the total cell number. Data are shown as mean ± SEM (n = 6). ***Indicates statistically significant values (P < 0.0001) vs. vehicle-treated cells. One way ANOVA, post-hoc Tukey’s. Full-length gels are showed in Supplementary Figs S6 and S7.

We therefore examined the effect of a 24 hour incubation of rat microglia with 1, 10, and 100 nM PEA on CB2R expression. IFNγ (300 U/mL for 24 hours) was used as a positive control^23^. No difference between untreated or vehicle-treated cells (0.01% DMSO) was observed (not shown). We found that the highest concentration of PEA (100 nM) significantly increased CB2 mRNA and protein levels in microglia compared to the levels in vehicle-treated cells (Fig. 1A and B). The involvement of PPAR-α in the PEA-mediated effect was evaluated by assessing the ability of the synthetic PPAR-α selective agonist GW7647 to modulate CB2R expression. CB2R expression was significantly higher in microglia treated with GW7647 (1 µM for 24 hours) than in vehicle-treated cells (Fig. S1A). Pre-treatment (15 min before) with the PPAR-α antagonist GW6471 (10 µM for 24 hours) completely blocked the enhancement of CB2R protein expression induced by both PEA and GW7647 (Figs 1B and S1A). The treatment with GW6471 alone did not significantly change CB2R expression, as compared with the vehicle (Fig. 1B). By immunocytochemical analysis, we also showed CB2R expression in cultured microglia. In particular, we observed the expression of CB2R in the cytosol of microglia incubated with PEA, GW7647 or IFNγ, whereas little staining was detected in vehicle-treated cells (Fig. 1C). Likewise, we found the presence of PPAR-α in the cytosol and, as punctate staining, also in the perinuclear area of resting or treated microglia (Fig. 1D).

Finally, we observed that all treatments induced morphological changes that are typically associated with an activated microglial state, such as increased soma diameters and shorter process lengths^24^. In contrast, vehicle-treated cells were in a ‘resting’ condition (Fig. 1F).

To investigate the involvement of PPAR-α in PEA effect on CB2R expression, we silenced *PPAR*-α mRNA expression in microglia and measured CB2R expression in the presence of PEA. Silencing *PPAR*-α gene expression with a specific siRNA led to a significant reduction in PPAR-α expression (81% reduction), whereas a non-targeting siRNA did not affect *PPAR*-α gene expression (Fig. 1E’). The receptor modulation observed in cells treated with the non-targeting siRNA was comparable to that observed in the absence of any siRNA treatment. In *PPAR*-α-silenced cells, incubation with PEA or GW7647 failed to increase CB2R expression, as compared to the levels of CB2R in cells treated with a non-targeting siRNA (Fig. 1E). CB2R antibody specificity was assessed in presence of the relative blocking peptide by western blotting and immunocytochemistry (Fig. S1B and C).

### PEA increases CB2R expression in a human macrophage cell culture

Unable to investigate microglia in humans, we explored the possibility of PEA-induced CB2R modulation in human macrophages, based on the evidence that resident microglia and blood-derived-macrophages are derived from the same cell lineage. Similar to the results from our microglia studies, we observed that under basal conditions (untreated or vehicle-treated cells), macrophages expressed low CB2R levels and treatment with PEA (100 nM) significantly increased both CB2 mRNA and protein expression (Fig. S2A and B). In particular, we observed a cyclic expression of mRNA transcripts in 2 and 12 hours-treated cells which was correlated with increased protein levels detected at 4 hours and possibly in the time interval between the 12 and 24 hours.

Treatment with GW7647 (1 µM for 4 hours) significantly enhanced CB2R expression and this effect was inhibited by GW6471 pre-treatment (10 µM for 4 hours) (Fig. S2C). Interestingly, a decrease in IFNγ-induced CB2R expression was observed in the presence of GW6471, suggesting possible cross-talk between PPAR-α and the IFNγ pathway in CB2R modulation (Fig. S2C). The quantification of CB2R protein expression was complemented by a double immunofluorescence experiment through which we demonstrated a merged fluorescence signal of CB2R and vimentin-labeled macrophages (Fig. S2D).

### Cnr2 is directly regulated by PEA-mediated PPAR-α activation: bioinformatic analysis and chromatin immunoprecipitation assay

In view of the PEA-induced increased levels of CB2R mRNA and protein observed in microglia, we explored the possibility of a direct regulation of the transcription of *Cnr2* (the gene encoding for CB2R) by PPAR-α. As nuclear receptors, PPARs forms heterodimers with the retinoid X receptor (RXR)^25^. Therefore, we used bioinformatic tools to search for putative consensus sequences for PPARα/RXR within the *Cnr2* gene, as well in its upstream untranslated 3′ and 5′-UTR region.

**Figure 2:**
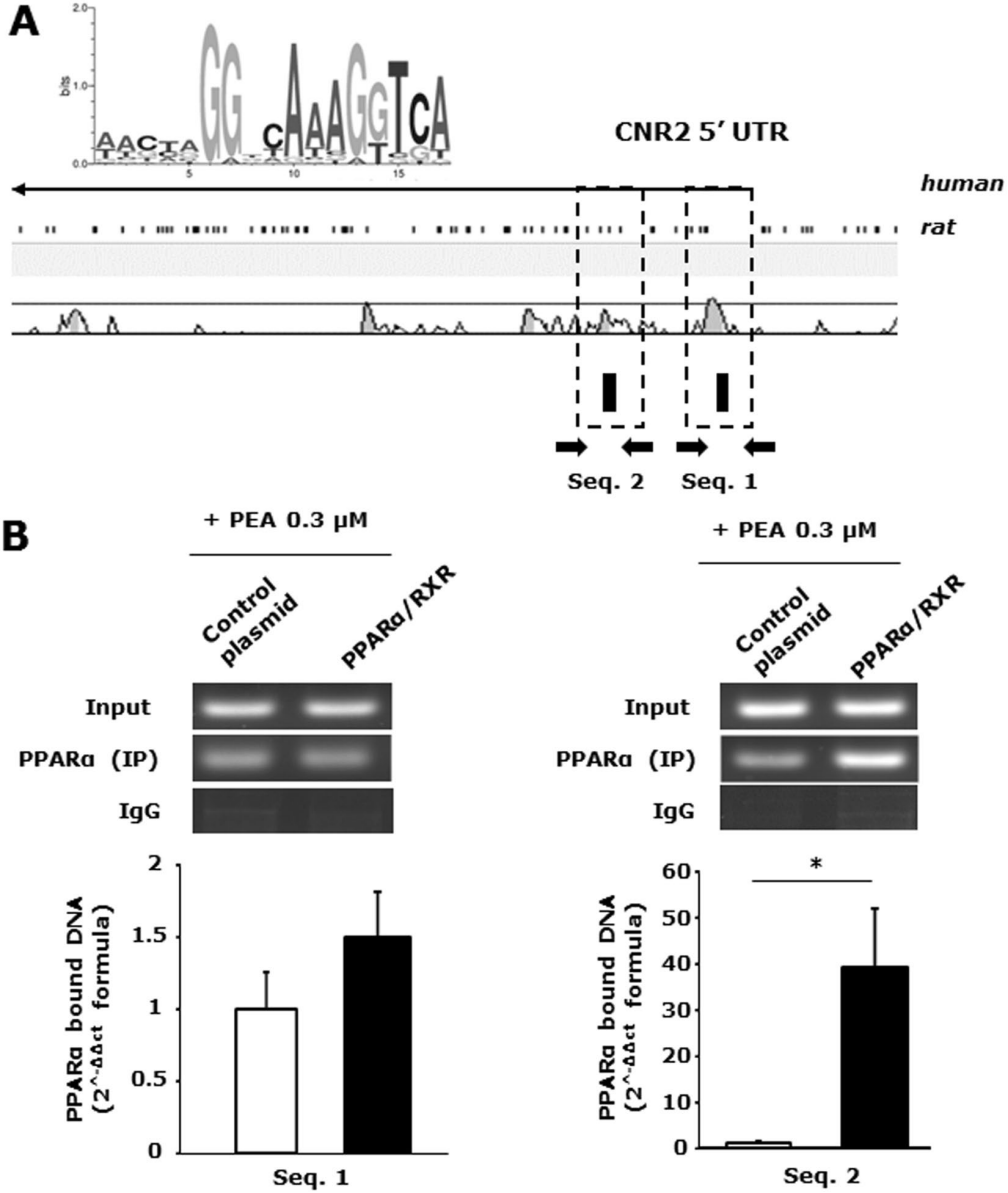
Cnr2 is directly regulated by PEA-mediated PPAR-α activation: bioinformatic analysis and chromatin immunoprecipitation assay. (**A**) A schematic alignment of the human and rat *Cnr2*. The black boxes correspond to regions of high sequence homology between the two species contaning the putative PPARα/RXR sites indicated as small black rectangles in the lower part below the schematic. The PPARα/RXR matrix used for the analysis is also shown in the upper part. (**B**) Average data of the relative amount of the PPARα -immunoprecipitated DNA in HEK293 cells transfected with either control (pCDNA3) or murine PPARα and RXR encoding plasmids. Data are from three to six separate experiments and normalized relative to the input DNA, using the 2^−ΔΔCt^ formula. The inset shows a representative agarose gel electrophoresis of the qPCR products obtained from PAX7-immunoprecipitated DNA for each experimental condition. Data are shown as mean ± SEM (n = 3) of three independent experiments conducted in triplicate. *p value ≤ 0.05 vscontrol condition (pcDNA-transfected cells), obtained using the unpaired T-TEST for statistical analyses.

The *Cnr2* sequence shows many differences between the two species^26^, and for this reason we searched for putative PPARα/RXR sequence-rich sites located within conserved regions, and identified putative binding sequences within two conserved regions located at the 5′UTR region of *Cnr2* (named sequence 1 and 2, Fig. 2A). Besides the two conserved regions identified at 5′UTR portion of the *Cnr2*, we found other putative PPARα/RXR sequences in non-conserved regions of both human and rat CB2.

Chromatin immunoprecipitation (Chip) analysis was performed to prove that, once stimulated with PEA, the nuclear receptor PPARα/RXR effectively binds the putative responsive sequences identified within *Cnr2*. Human embryonic kidney (HEK293) cells were transiently transfected with control (pcDNA3) or PPARα/RXR encoding plasmids. After 24 hours, transfected HEK293 cells were treated with PEA (0.1 or 0.3 µM for 24 hours). Afterwards, the genomic DNA was isolated from control and PPARα/RXR transfected cells. Then, the DNA was sonicated to obtain fragments between 400–500 bp and used for the immune-affinity reaction using a specific anti-PPAR-α antibody (see experimental procedure section). Subsequently, PPAR-α-immunoprecipitated DNA fragments were used for qPCR analysis using a specific pair of primers able to amplify the genomic portions of interest within the *Cnr2* sequence containing the putative PPAR-α binding sites (black arrows, Fig. 2A). We found that upon stimulation with PEA 0.3 µM, PPAR-α binds with high affinity to only one of the two conserved regions identified in *Cnr2* (Fig. 2B). Furthermore, we found a similar result upon exposure of PPARα/RXR transfected HEK293 cells to PEA 0.1 µM, although to a lesser extent, thus suggesting a plausible dose dependent effect exterted on PPARa/RXR heterodimerization/activation (data not shown).

### PEA enhances phagocytosis and intracellular killing of P. gingivalis by microglial cells

**Figure 3:**
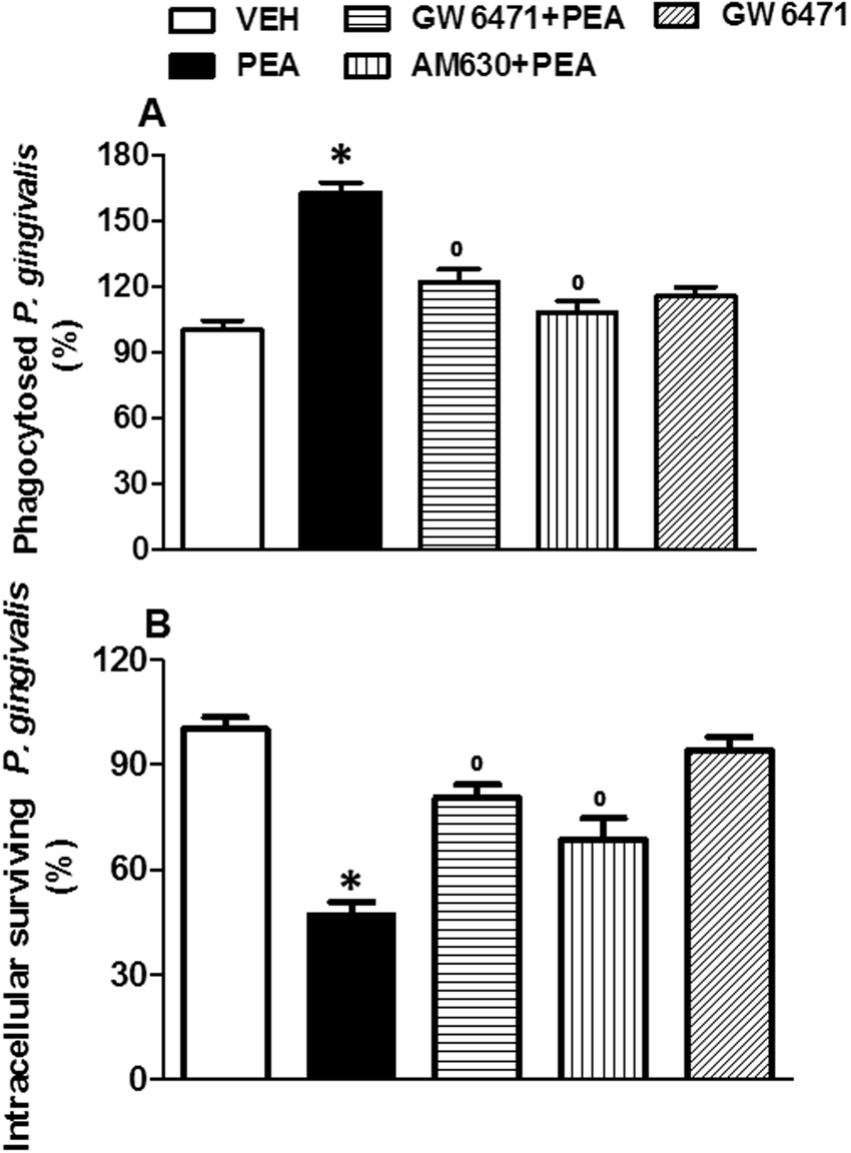
PEA enhances phagocytosis and intracellular killing of *P. gingivalis* by microglial cells. Phagocytosis assay (**A**) and intracellular survival evaluation (**B**) were performed in the presence of PEA (100 nM), or GW6471 (10 µM) + PEA, or AM630 (100 nM) + PEA, or GW6471 alone. The number of bacteria ingested (at 90 min) or number of bacteria recovered (at 270 min) by the control group (vehicle) were considered to be 100%. Data are shown as means ± SEM (n = 6). *P < 0.05 compared to vehicle, °P < 0.05 compared to PEA. One way ANOVA, post-hoc Tukey’s.

To assess the effect of PEA on the phagocytosis of *P. gingivalis* by microglia, cells were cultured with *P. gingivalis* and incubated with PEA or vehicle. No significant difference was observed between untreated and vehicle-treated cells (not shown). The numbers of ingested *P. gingivalis* after 24 hours of PEA treatment are shown in Fig. 3. We observed that PEA (100 nM) induced a significant enhancement of ingestion of bacteria as compared to vehicle-treated cells (Fig. 3A). In particular, in cells pretreated with PEA, the amount of phagocytosed *P. gingivalis* was 60% higher than that in control. When blocking PPAR-α with GW6471 (10 µM), the phagocytic activity was normalized; similar results were obtained by blocking CB2R (AM630, 100 nM) (Fig. 3A). Additionally, we observed that PEA treatment resulted in a decreased number of surviving *P. gingivalis* after 4 hours of incubation compared to the control (~46%). The pretreatment with GW6471 or AM630 significantly reduced PEA-mediated effects compared to untreated cells (Fig. 3B). GW6471 alone (10 µM) did not change the phagocytosis or intracellular killing of *P. gingivalis* as compared with vehicle (Fig. 3A and B).

### PEA induces microglia migration through CB2R

**Figure 4:**
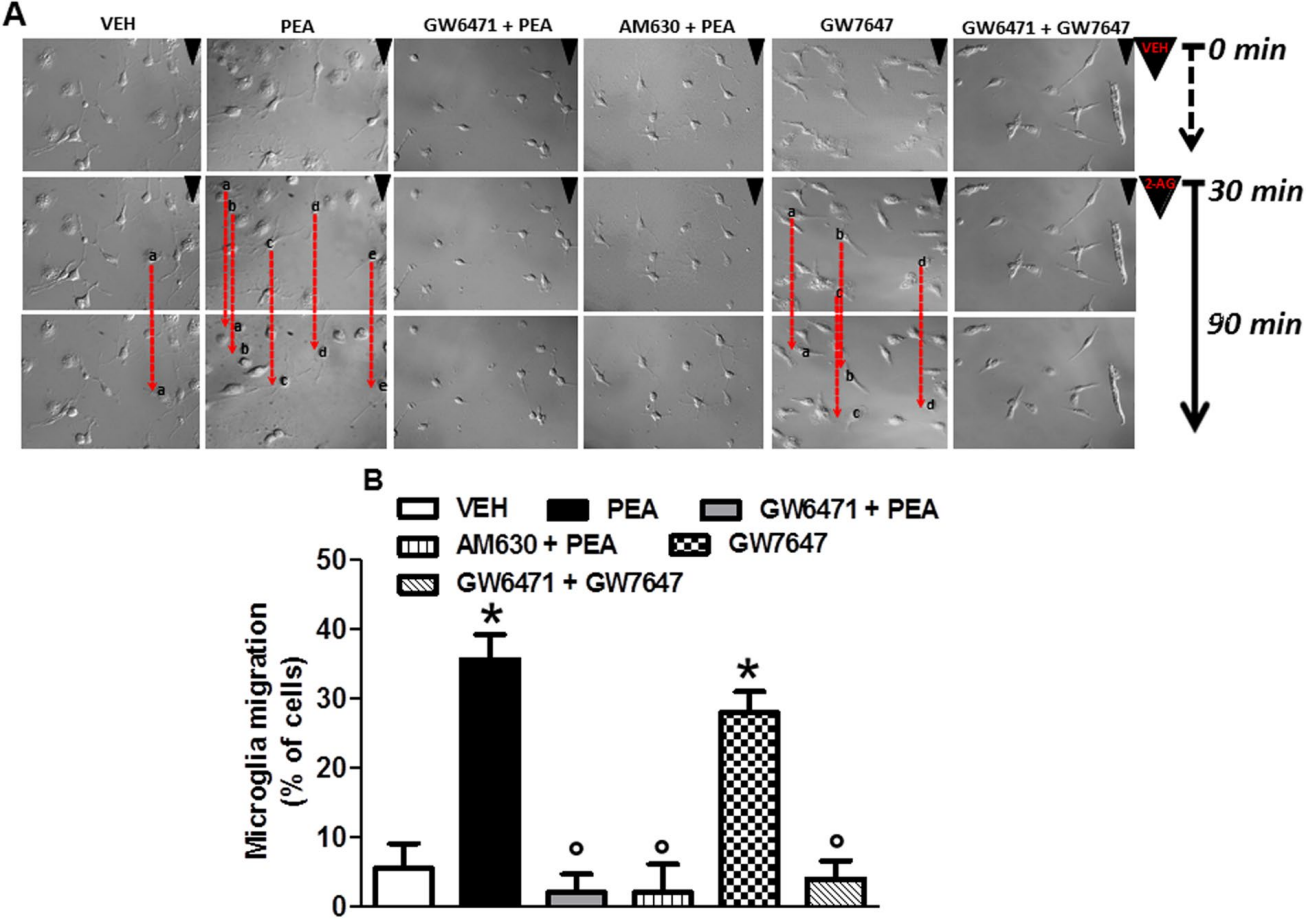
PEA induces microglia migration towards a 2-AG source. (**A**) Image-based detection of 2-AG (100 µM) chemoattractive effect on cultured microglial cells after different treatments. Panel shows the migratory effect induced by PEA (100 nM) or GW7647 (1 µM), alone or in the presence of GW6471 (10 µM) or AM630 (100 nM), as compared to the control group (vehicle). Representative data of microglial cell time-lapse migration recorded at starting point (0 min), 30 and 90 min from the 2-AG exposure. (**B**) The quantification indicates the percentage of microglia cells affected by 2-AG chemoattractive movements vs total cell number at 90 min. Data are shown as means ± SEM (n = 6–8). *P < 0.05 compared to vehicle and °P < 0.05 compared to PEA or GW7647. One way ANOVA, post-hoc Tukey’s. Scale bar = 50 µm.

To assess the possible capability of PEA to induce and/or potentate microglia motility/migration we used a time-lapse experiment in which microglia migrate towards a source of 2-arachidonoylglycerol (2-AG) [100 µM, one of the two major eCBs]. Minimum microglial migration was observed under physiological conditions. PEA incubation (100 nM for 24 hours) significantly increased the migratory activity as compared to vehicle-treated cells. Interestingly, the pre-treatment with AM630 (100 nM) or GW6471 (10 µM) blocked 2-AG-induced chemotaxis, confirming the involvement of CB2R and PPAR-α in this process. In a similar fashion to PEA, the synthetic PPAR-α agonist also improved microglial migration, suggesting that the cannabinoid-induced microglial chemotaxis through CB2R could be selectively mediated by PPAR-α (Fig. 4). Conversely, we observed that pre-treatment with the CB1R antagonist AM251 (500 nM for 24 hours) did not modify PEA-mediated migration (Fig. S4).

### Microglial cells produce sufficient amounts of 2-AG to tonically activate the PEA-induced upregulated CB2R in the presence of P. gingivalis

Next, we measured by isotope-dilution LC-MS the amounts of 2-AG produced by microglial cells stimulated with vehicle, *P. gingivalis* and *P. gingivalis* plus PEA (100 nM). We found that levels of 2-AG were much higher than those of the other endocannabinoid, anandamide, and potentially sufficient to tonically activate CB2R (39.6 ± 8.0 *vs.* 1.2 ± 0.2 pmol/mg lipid extract, equivalent to 17.9 ± 3.4 *vs.* 0.5 ± 0.06 nM in 1 ml of culture, means ± SD, n = 3; each ml of culture contained 5 × 10^5^ cells), particularly if these receptors are upregulated (Fig. S5). Following *P. gingivalis* or *P. gingivalis* plus PEA incubation, we found that 2-AG levels were not significantly increased (up to 53.6 ± 12.7 and 74.04 ± 12.7 pmol/mg of extracted lipids, respectively, means ± SD, n = 3), whereas anandamide levels were significantly increased only following co-incubation with *P. gingivalis* plus PEA (up to 1.0 ± 0.16 and 2.7 ± 0.2 pmol/mg of extracted lipids, respectively, means ± SD, n = 3). Importantly, PEA was also detected and abundant in microglia under our experimental conditions (20.1 ± 2.3 and 17.6 ± 5.6 pmol/mg of extracted lipids, respectively in vehicle and *P. gingivalis*-stimulated cells, means ± SD, n = 3, equivalent to 9.2 ± 1.3 and 6.1 ± 1.8 nM, respectively, per 1 ml of cultured cells) (Fig. S5).

## Discussion

The present study adds another piece to the intricate mosaic of the proposed mechanisms for the pleiotropic effects of PEA. In particular, we show that this endogenous fatty acid ethanolamide affects cannabinoid signaling by upregulating CB2R expression in mononuclear phagocytic cells. A significant increase in CB2R expression was observed in cultured rat microglia and human macrophages following incubation with PEA. Pharmacological, biochemical and computational analyses revealed that CB2R upregulation occurs through a PPAR-α-mediated genomic mechanism. Furthermore, we provide evidence for the involvement of CB2R in PEA-induced microglial phagocytosis and migration *in vitro*.

Previous evidence suggests a role for CB2R in PEA-mediated effects under different pathological conditions with particular regard to chronic pain states^19, 20, 27^. PEA, which does not directly activate CB1R or CB2R^28^, shows anti-inflammatory properties that can be blocked by the selective CB2 antagonist SR144528^22^. These findings have suggested that these PEA-mediated effects may result from direct stimulation of yet uncharacterized CB2-like receptors. Moreover, an ‘entourage hypothesis’, which states that the effects of PEA are due to enhancement of eCBs, and thus are a consequence of CBR or TRPV1 stimulation, has been also proposed^29^. However, the mechanism by which PEA interacts with CB2R is unknown. Here we show, for the first time, that PEA increases CB2R expression through a genomic mechanism involving the activation of the best established direct target for this lipid compound, i.e. PPAR-α. Because selective stimulation of CB2R is associated with analgesia and the release of anti-inflammatory cytokines^30–32^, our findings may explain the involvement of CB2R in the therapeutic effects of PEA, which are often observed in pathologies involving specific immune responses. In fact, the presence of CB2R in immune cells, including microglia, has been conclusively established^6^. Microglia closely resemble tissue macrophages and are key sensors of CNS injury caused by different diseases^33–37^. They are known to rapidly proliferate under activation and exert both pro- and anti-inflammatory effects^38^ in the inflammatory response. Milligan and Watkins^39^ suggested that the neuroprotective microglia (M2 phenotype) overexpress both CB1R and CB2R, which concurrently control the release of pro- and anti-inflammatory cytokines. However, the role of CBRs in microglia physiology has not been fully clarified. Here, we show that microglia increase CB2R expression following PEA treatment under physiological conditions, thereby assuming a hypertrophic morphology. In fact, using IFNγ as a positive control^23^, we found that treatment with PEA significantly increased CB2R levels in microglia, in association with cytoskeletal rearrangements that are typical of morphological cell activation. Indeed, as shown by immunofluorescence, PEA-treated cells exhibited larger somata (diameter) and shorter processes than control cells. A stringent analysis of surface antigens may identify possible changes in the M1/M2 cell profile induced by PEA.

The involvement of PPAR-α in the effects of PEA was evaluated by assessing the ability of the synthetic PPAR-α-selective agonist GW7647 to up-regulate CB2R. We found that GW7647 significantly increases CB2R expression levels compared to those in non-treated cells, and pre-treatment with the PPAR-α antagonist GW6471 blocks this effect. The requirement for PPAR-α in PEA-induced CB2R up-regulation was shown by silencing PPAR-α mRNA. Indeed, in PPAR-α-silenced microglia (which exhibit a ~80% reduction in PPAR-α mRNA levels), incubation with PEA or GW7647 failed to increase CB2R expression.

Our results suggest that the mechanism underlying PEA-induced CB2R up-regulation involves the activation of PPAR-α. To obtain conclusive evidence for this hypothesis, we performed ChiP assay by transfecting PPAR-α in HEK293 cells, a common cell model used to perform heterologous expression studies^40^, and evaluated the possible induction of a physical interaction between PPAR-α and *Cnr2* by PEA. We found that upon stimulation with PEA, PPAR-α binds with high affinity a specific region identified in the *Cnr2* gene. Thus, our results clearly show a straight gene regulation operated by PEA, although this drug could modulate several other pharmacological targets. Interestingly, compared with microglia, a higher dose of PEA (300 nM) was necessary to induce appreciable changes in HEK293 cells. This could be explained by assuming that primary microglia contain constitutively a more efficacious PPAR-α signaling system.

Unable to investigate resident microglia cells in humans, we decided to evaluated possible the PEA-induced CB2R modulation in blood-derived human macrophages, because of the similarity of the derived-cell lineage between microglia and macrophages. Indeed, similar to microglia, in human macrophage cells cultures we also observed that PEA and GW7647 increased CB2R expression. However, in these cells, the enhancement of protein expression was observed after a shorter incubation period (4 hours) than that observed in microglia (24 hours). This different time course of CB2R expression might be consequence of the different cell type used (human macrophages *vs* rat microglia) and, hence, of the possible different PPAR-α expression levels, as well as, localization/expression of the CB2R protein. Interestingly, decreased IFNγ-induced CB2R expression was observed in the presence of the PPAR-α antagonist, suggesting the existence of a cross-talk between the PPAR-α and IFNγ pathways in CB2R modulation in human macrophages.

In agreement with its anti-inflammatory/neuroprotective activities, previous studies proved the capability of PEA to increase resistance against systemic infections *in vivo* ^41–44^. Indeed, short-term PEA exposure (30 min) stimulates *E. coli* uptake in macrophages and microglia^45–47^. Our findings confirm the ability of PEA to increase microglia phagocytosis of the periodontal *P. gingivalis* pathogens. We have choosen *P. gingivalis* because these bacteria or their virulent factors are not only a source of chronic infections and inflammation, but can also reach the brain systemically over time and, by priming the resident immune cells (such as microglia), trigger neuroinflammatory processes associated with early stages of dementia^47^. In this study, we showed that treatment with PEA increases phagocytosis and enhances intracellular bacteria killing by microglia. Moreover, we demonstrated that the effects of PEA were mediated by both PPAR-α and CB2R activation. Our findings suggest that PEA or CB2R agonists may prevent some forms of brain infection, and, consequently, inflammatory neurodegeneration, by promoting microglial phagocytosis. Conversely, a recent study by Redlich and colleagues (2012)^46^ showed that the ability of PEA to induce phagocytosis does not depend on PPAR-α stimulation. This discrepancy may be due to the different bacterial strains (*Streptococcus pneumonia* or *E. coli vs P. gingivalis*) and cell lines (mouse vs rat) used in the two studies. Additionally, prolonged PEA treatment might promote CB2R activation, as shown here and elsewhere, also by merely enhancing eCB levels^48^, and thus induce phagocytosis through a PPAR-α-independent mechanism. At any rate, the present findings provide for the first time a molecular mechanism for PEA capability to increase resistance against systemic infections, an effect that has been known for decades and has been already exploited in the clinic in the past when PEA was marketed as “impulsin”^49^.

The recruitment of phagocytic cells to sites of damage is a crucial step in inflammation and antimicrobial immune responses. Microglia cell migration can be triggered by a pool of chemoattractant factors acting at G-coupled receptors, which include chemokines, nucleotides, and bioactive lipids (i.e. eCBs)^50–53^. In this context, CB receptor modulators (synthetic compounds or phytocannabinoids) have been shown to regulate macrophage/microglial cell activity and migration in several conditions^54–57^. Our present data reveal that the increased CB2R expression mediated by PEA manifests also an enhanced capability of microglia to migrate towards a high (μM) local concentration of extracellular 2-AG source. On the other hand, we found that, following stimulation with PEA and *P. gingivalis*, lower (nM) concentrations of 2-AG are present in microglia, which, alone or together with the other eCB, anandamide, might activate the PEA-upregulated CB2R and trigger phagocytosis, but would not be sufficiently high to prevent microglia migration towards an external source of eCBs. Indeed, although it is always difficult to predict to what extent the global concentrations of very lipophilic compounds (such as the eCBs and PEA) that are detected in an experimental set-up reflect the local concentration at their targets (CBRs and PPAR-α, respectively) in a biological setting, we can surmise, from our present findings and previous data on its functional activity at CB2R^58, 59^, that only microglial 2-AG might tonically stimulate the CB2R upregulated by PEA, and that also PEA coming from neighboring cells is necessary to exert the upregulation of CB2R.

In conclusion, the present study provides evidence for a new mechanism of action for PEA. Our data indicate that PEA may act indirectly on CB2R, not only by increasing endogenous ligand content^29, 60^, but also by regulating receptor expression. Moreover, we demonstrated that PEA induces a microglial morphological change associated with enhanced phagocytosis and migratory activity in which CB2R plays a role. These findings suggest that PEA, by targeting CB2R, may be a useful tool for preventing the consequences of neuroinflammation in CNS disorders, and explain the clinical use in the late 1970’s of this compound against infections.

## Experimental Procedures

### Cell cultures and transfection

-Microglial Cells. Primary microglia were prepared according to our previous studies^61, 62^ (Supplemental Experimental Procedures). Briefly, cortices from SD postnatal rats were dissociated and suspended in Dulbecco’s modified Eagle medium (DMEM). The cell suspension was filtered and plated in tissue culture flasks precoated with poly-D-lysine. Cells were harvested as floating cell suspensions following shaking after 10–12 days. Cell viability was determined by measuring the lactate dehydrogenase (LDH assay kit, Sigma, UK) according to the manufacturers’ instructions. In all experiments no significant difference was observed between untreated and vehicle-treated cells (DMSO 0.01%). The experimental procedures were conducted in conformity with protocols approved by the Animal Ethics Committee of the Second University of Naples. Animal care was in compliance with the IASP and European Community (E.C. L358/1 18/12/86) guidelines on the use and protection of animals in experimental research.

- Human Embryonic Kidney-293 (HEK293) cells were grown in 60-mm plastic Petri dishes in Eagle’s Minimum Essential Medium (EMEM) containing 10% FBS, nonessential amino acids (0.1 mM), penicillin (50 U/ml), and streptomycin (100 µg/ml) in a humidified atmosphere at 95% O_2_, 5% CO_2_ at 37 °C. After plating, the cells were transfected on the next day with plasmids encoding murine PPAR-α (Addgene, Plasmid #22751) and retinoid X receptor (RXR Addgene, Plasmid #8882) cDNAs by use of Lipofectamine LTX (Life Tecnology) following manufacturer’s instructions. One day after transfection, PEA was added to the culture medium for 24 hours.

- Macrophages. Eight healthy volunteers were enrolled as whole blood donors for macrophages cultures after written informed consent with the approval of the Ethic Committee of the Second University of Naples (Supplemental Experimental Procedures).

All procedures were carried out in accordance with the national legislation and the Declaration of Helsinki.

### Immunocytochemistry

Cells were fixed in 4% paraformaldehyde for 20 min, followed by ice-cold 100% methanol for 3 min. Fixed cells were incubated for 2 h with primary antibodies against Iba-1 (rabbit anti-ionized calcium binding adapter molecule 1; 1:1000 dilution; Wako Chemicals, Germany), or Vimentin (mouse anti-Vimentin; 1:500 dilution; Santa Cruz Biotechnology), CB2R (goat anti-CB2 1:200 dilution; Santa Cruz Biotechnology) or PPAR-α (mouse anti PPAR-α 1:400 diluition, Abcam). Following this incubation, cells were washed and incubated for 45 min with the secondary antibody solution (donkey anti-rabbit or donkey anti-goat or donkey anti-mouse IgG-conjugated Alexa Fluor TM 488 and 568; 1:1000 dilution; Molecular Probes). Coverslips were mounted using Vectashield mounting media (Vector Laboratories, Burlingame, CA). Activated cells were detected by measuring the cell soma and processes length. Resting and activated microglia were classified based on the following criteria: resting microglia displayed small somata bearing long, thin, ramified processes, whereas activated microglia exhibited marked cellular hypertrophy and retraction of processes such that the process length was lower than the diameter of the soma compartment. Data were expressed as percentage of activated cells. All experiments were performed in triplicate.

### Real Time Polymerase Chain Reaction

To quantify the expression levels of CB2R mRNA, three serial complementary DNA (cDNA) dilutions (1:5) obtained from reverse transcription of 100 ng total mRNA (High Capacity cDNA Reverse Transcription kit; Applied Biosystems, Foster City, CA, USA) were amplified by Real Time polymerase chain reaction (Real Time PCR or qPCR) with SYBR green as fluorophore. The housekeeping gene β-actin was used as endogenous control. Real Time-PCR products were analyzed using the SDS software (Applied Biosystems, Foster City, CA, USA) (Supplemental Experimental Procedures).

### RNA extraction and RT-PCR

Total RNA was extracted from homogenized cells using a RNA Tri-Reagent (Molecular Research Center Inc., Cincinnati, OH) according to the manufacturer’s protocol. The extracted RNA was subjected to *DNase* I treatment at 37 °C for 30 min. The total RNA concentration was determined by UV spectrophotometry. The mRNA levels of the genes under analysis were measured by RT-PCR amplification. RT minus controls were carried out to check potential genomic DNA contamination. These RT minus controls were performed without using the reverse transcriptase enzyme in the reaction mix. Sequences for the mRNAs from GeneBank (DNASTAR INC., Madison, WI) were used to design primer pairs for RT-PCRs (OLIGO 4.05 software, National Biosciences Inc., Plymouth, MN). Each RT-PCR was repeated at least three times to achieve the best possible reproducibility data. The measured mRNA levels were normalized with respect to hypoxanthine-guanine phosphoribosyltransferase (HPRT), chosen as housekeeping gene. The HPRT gene expression did not change in several experimental conditions. To our knowledge there is no molecular evidence for variation in HPRT mRNA-levels in this model. The gene expression values were expressed as arbitrary units ± SE. Amplification of genes of interest and HPRT were performed simultaneously. PCR products were resolved into 2.0% agarose gel. A semi-quantitative analysis of mRNA levels was carried out by the Gel Doc EZ UV System (Bio-Rad, Hercules, CA).

### Protein Extraction and Western Blotting Analysis

For protein extraction, cells or tissues were collected in RIPA buffer (Thermo Fisher Scientific Inc., Rockford, IL, USA), and proteins were extracted according to the manufacturer’s protocol. Protein concentrations were determined using the Bradford reagent (Sigma-Aldrich, St Louis, MO, USA). Samples were run on a 12% or 15% sodium dodecyl sulfate–polyacrylamide gel and transferred to nitrocellulose membranes. Membranes strips were blocked in 5% milk, 13 Tris-buffered saline, and 0.05% Tween-20 and incubated, firstly overnight with primary antibodies to detect CB2R (1:200; Abcam, Cambridge, UK) or PPARα (PPAR-α 1:400 diluition, Abcam) and then with the relative secondary antibody. A blocking peptide for the anti-CB2R antibody (1:100 diluition, Abcam) in control microglia cells or spleen was used to validate the specificity of the antibody. An anti-β-tubulin monoclonal antibody (1:5000 dilution; Santa Cruz Biotechnology Santa Cruz, CA, USA) was used to check for identical protein loading. Reactive bands were detected by chemiluminescence (SuperSignal, West Femto, Pierce, USA) and visualized on a X-ray film (Fuji Corporation, Tokyo, Japan) or on a C-DiGit® Blot Scanner (LI-COR Biosciences, Nebraska, USA) or on a ChemiDoc station (Bio-Rad, Hercules, CA, USA). Images were captured, stored, and analyzed using “Image studio Digits ver. 5.0” software.

### Gene silencing

PPAR-α mRNA was silenced by using a human PPAR-α siRNA reagent (PPAR-a silencer select Pre-designed siRNA, Ambion, USA). Cell transfection was performed according to manufacturer’s protocol. Briefly, microglial cells were obtained as described above and plated on 24-multiwell plates with D-MEM and 10% FBS at density 500.000 cells/well. Cells were then transfected by using 1 μM non-targeting or experimental siRNA combined in 1%-supplemented FBS medium. Cells were incubated at 37 °C in a CO_2_ incubator for 3 days, after which they were collected for bio-molecular examination or treated with PEA (100 nM), GW7647 (1 μM), and IFNγ (300 U/mL) for 24 hours in order to evaluate CB2R protein expression. Transfection minus controls were carried out using non-targeting siRNA in order to check the efficiency of transfection.

### Bioinformatic analysis and chromatin immunoprecipitation assays

Putative PPAR/RXR consensus sequence identification was performed by using Genomatix software. The ChIP assay was performed by the use of ChiP Kit (Sigma-Aldrich MI, IT; cat. n. CHP1) following the manufacturer’s instructions. The genomic DNA was sonicated, immunoprecipitated with an anti-PPARα antibody certified for ChiP analysis (Abcam; ab2779) and subsequently released from histones by following the previously described procedure^63^. Immunoprecipitated DNA was then analyzed by qPCR (40–45 cycles) using specific primers able to amplify each of PPAR/RXR-rich regions identified (Forward: GTC AGG TAC CTC TCA GCT CC; Reverse CTG TAA GGG GAT AAT GCA CC the sequence 1; Forward: GGT GCA TTA TCC CCA TTT TAC AG; Reverse: GTG AGT GTG AGG AGG TCT GG for the sequence 2). In addition, for each primer pair, non-immunoprecipitated DNA was used as input for normalization. All ChIP assays were performed in triplicate for at least three different biological preparations (Fig. S3).

### Phagocytosis assay and intracellular survival assay

Pretreated microglia with PEA (100 nM), and/or GW6471 (10 µM), and/or AM630 (100 nM) for 24 hours were infected with *P. gingivalis* cells. Ingested bacteria were serially diluted and spread on plates for viable counts. To monitor survival, microglial cells were incubated with *P. gingivalis* for 90 min. Thereafter, cells were washed and the extracellular bacteria destroyed. The amounts of intracellular bacteria were determined as CFU by quantitative plating of serial dilutions on TS agar (Supplemental Experimental Procedures).

### Migration assay

The migration assay was performed in PEA or PPAR-α agonist-treated cells, in presence of the PPAR-α selective antagonist GW6471 (10 µM) or the CB2R selective antagonist AM630 100 nM), or the CB1 selective antagonist AM251 (500 nM). The movement of microglia was recorded each 5 min for 90 min starting from the application of 2-AG as the chemo-attractant ligand (Supplemental Experimental Procedures).

### Quantification of eCBs and related mediators in microglial cells

Cell samples were dounce-homogenized and extracted in 5 vol of chloroform/methanol/Tris–HCl 50 mM (2:1:1) containing 5 pmol of [^2^H]_8_ anandamide and 50 pmol of [^2^H]_4_ palmitoylethanolamide (PEA) and [^2^H]_5_ 2-AG (Cayman Chemicals, MI, USA). The lipid-containing organic phase was dried down in a rotating evaporator, weighed, and pre-purified by open-bed chromatography on silica gel. Lyophilized extracts were resuspended in chloroform/methanol 99:1 by vol. The solutions were then purified by open bed chromatography on silica as described^64^. Fractions eluted with chloroform/methanol 9:1 by vol. (containing 2-AG, anandamide and PEA) were collected and the excess solvent evaporated with a rotating evaporator, and aliquots analyzed by isotope dilution-liquid chromatography/atmospheric pressure chemical ionisation/mass spectrometry (LC-APCI–MS) carried out under conditions described previously^65^ and allowing the separations of 2-AG, anandamide and PEA. MS detection was carried out in the selected ion monitoring mode using m/z values of 356 and 348 (molecular ion +1 for deuterated and undeuterated anandamide), 384.35 and 379.35 (molecular ion +1 for deuterated and undeuterated 2-AG), 304 and 300 (molecular ion +1 for deuterated and undeuterated PEA). The amounts of all mediators were expressed as pmol/mg of cells.

## Electronic supplementary material

Supplementary Information

## References

[1] Kettenmann H, Hanisch U-K, Noda M, Verkhratsky A (2011). Physiology of microglia. Physiol. Rev..

[2] McMahon SB, Malcangio M (2009). Current challenges in glia-pain biology. Neuron.

[3] Franco R, Fernàndez-Suàrezs D (2015). Alternatively activated microglia and macrophages in the central nervous system. Prog. Neurobiol..

[4] Sierra A, Tremblay ME, Wake H (2014). Never-resting microglia: physiological roles in the healthy brain and pathological implications. Front. Cell. Neurosci.

[5] von Bernhardi R, von Bernhardi Eugenín L, Eugenín. (2015). Microglial cell dysregulation in brain aging and neurodegeneration. Front Aging Neurosci.

[6] Stella N (2010). Cannabinoid and cannabinoid-like receptors in microglia, astrocytes, and astrocytomas. Glia.

[7] Luongo L (2010). 1-(2′,4′-dichlorophenyl)−6-methyl-N-cyclohexylamine-1,4-dihydroindeno[1,2-c]pyrazole-3-carboxamide, a novel CB2 agonist, alleviates neuropathic pain through functional microglial changes in mice. Neurobiol. Dis..

[8] Romero-Sandoval EA, Horvath R, Landry RP, DeLeo JA (2009). Cannabinoid receptor type 2 activation induces a microglial anti-inflammatory phenotype and reduces migration via MKP induction and ERK dephosphorylation. Mol Pain..

[9] Ehrhart J (2005). Stimulation of cannabinoid receptor 2 (CB2) suppresses microglial activation. Neuroinflammation.

[10] Facci L (1995). Mast cells express a peripheral cannabinoid receptor with differential sensitivity to anandamide and palmitoylethanolamide. Proc. Natl. Acad. Sci. USA.

[11] Scarampella F, Abramo F, Noli C (2001). Clinical and histological evaluation of an analogue of palmitoylethanolamide, PLR 120 (comicronized Palmidrol INN) in cats with eosinophilic granuloma and eosinophilic plaque: a pilot study. Vet. Dermatol..

[12] Skaper SD, Facci L, Giusti P (2013). Glia and mast cells as targets for palmitoylethanolamide, an anti-inflammatory and neuroprotective lipid mediator. Mol. Neurobiol..

[13] Mattace Raso G, Russo R, Calignano A, Meli R (2014). Palmitoylethanolamide in CNS health and disease. Pharmacol. Res..

[14] Koch M (2011). Palmitoylethanolamide protects dentate gyrus granule cells via peroxisome proliferator-activated receptor-α. Neurotox Res..

[15] Costa B, Comelli F, Bettoni I, Colleoni M, Giagnoni G (2008). The endogenous fatty acid amide, palmitoylethanolamide, has anti-allodynic and anti-hyperalgesic effects in a murine model of neuropathic pain: involvement of CB(1), TRPV1 and PPARgamma receptors and neurotrophic factors. Pain.

[16] Di Cesare Mannelli L (2013). Palmitoylethanolamide is a disease-modifying agent in peripheral neuropathy: pain relief and neuroprotection share a PPAR-alpha-mediated mechanism. Mediators Inflamm..

[17] Luongo L (2013). Palmitoylethanolamide reduces formalin-induced neuropathic-like behaviour through spinal glial/microglial phenotypical changes in mice. CNS Neurol. Disord. Drug Targets.

[18] Mackie K, Stella N (2006). Cannabinoid receptors and endocannabinoids: evidence for new players. AAPS J..

[19] Calignano A, La Rana G, Giuffrida A, Piomelli D (1998). Control of pain initiation by endogenous cannabinoids. Nat.

[20] Calignano A, La Rana G, Loubet-Lescoulié P, Piomelli D (2000). A role for the endogenous cannabinoid system in the peripheral control of pain initiation. Prog. Brain. Res..

[21] LoVerme J, La Rana G, Russo R, Calignano A, Piomelli D (2005). The search for the palmitoylethanolamide receptor. Life. Sci..

[22] Iannotti FA, Di Marzo V, Petrosino S (2016). Endocannabinoids and endocannabinoid-related mediators: Targets, metabolism and role in neurological disorders. Prog. Lipid. Res..

[23] Cabral GA, Marciano-Cabral F (2005). Cannabinoid receptors in microglia of the central nervous system: immune functional relevance. J. Leukoc. Biol..

[24] Hains B, Waxman S (2006). Activated microglia contribute to the maintenance of chronic pain after spinal cord injury. J. Neurol. Sci..

[25] Chandra V (2008). Structure of the intact PPAR-gamma-RXR- nuclear receptor complex on DNA Species differences in cannabinoid receptor 2 (CNR2 gene): identification of novel human and rodent. Nature.

[26] Liu QR (2009). Species differences in cannabinoid receptor 2 (CNR2 gene): identification of novel human and rodent CB2 isoforms,differential tissue expression and regulation by cannabinoid receptor ligands. Genes Brain Behav.

[27] Farquhar-Smith W-P, Rice AS (2003). A novel neuroimmune mechanism in cannabinoid-mediated attenuation of nerve growth factor-induced hyperalgesia. Anesthesiology.

[28] Showalter V, Compton DR, Martin BR, Abood ME (1996). Evaluation of binding in a transfected cell line expressing a peripheral cannabinoid receptor (CB2): identification of cannabinoid receptor subtype selective ligands. J. Pharmacol. Exp. Ther..

[29] Re G, Barbero R, Miolo A, Di Marzo V (2007). Palmitoylethanolamide, endocannabinoids and related cannabimimetic compounds in protection against tissue inflammation and pain: potential use in companion animals. Vet. J..

[30] Racz I (2008). Crucial role of CB(2) cannabinoid receptor in the regulation of central immune responses during neuropathic pain. J. Neurosci..

[31] Pasquini S (2012). Design, synthesis, and pharmacological characterization of indol-3-ylacetamides, indol-3-yloxoacetamides, and indol-3-ylcarboxamides: potent and selective CB2 cannabinoid receptor inverse agonists. J. Med. Chem..

[32] Mugnaini C (2012). Investigations on the 4-quinolone-3-carboxylic acid motif part 5: modulation of the physicochemical profile of a set of potent and selective cannabinoid-2 receptor ligands through a bioisosteric approach. Chem. Med. Chem..

[33] Luongo L (2012). 5′-Chloro-5′-deoxy-(±)-ENBA, a potent and selective adenosine A(1) receptor agonist, alleviates neuropathic pain in mice through functional glial and microglial changes without affecting motor or cardiovascular functions. Molecules.

[34] Guida F (2012). Salvinorin A reduces mechanical allodynia and spinal neuronal hyperexcitability induced by peripheral formalin injection. Mol. Pain..

[35] Guida F (2015). Palmitoylethanolamide reduces pain-related behaviors and restores glutamatergic synapses homeostasis in the medial prefrontal cortex of neuropathic mice. Mol. Brain.

[36] Rinaldi B (2015). Effect of Prolonged Moderate Exercise on the Changes of Nonneuronal Cells in Early Myocardial Infarction. Neural Plast..

[37] Spaziano, G. *et al.* Exposure to Allergen Causes Changes in NTS Neural Activities after Intratracheal Capsaicin Application, in Endocannabinoid Levels and in the Glia Morphology of NTS. *Biomed. Res.* 980983 (2015). 10.1155/2015/980983 PMC4383154 25866824

[38] Romero-Sandovalm EA, Horvathm RJ, De Leom JA (2008). Neuroimmune interactions and pain: focus on glial-modulating targets. Curr. Opin. Investig. Drugs..

[39] Milliganm E, Watkins L (2009). Pathological and protective roles of glia in chronic pain. Nat. Rev. Neurosci..

[40] Lin YC (2014). Genome dynamics of the human embryonic kidney 293 lineage in response to cell biology manipulations. Nat. Commun..

[41] Loria F (2008). Study of the regulation of the endocannabinoid system in a virus model of multiple sclerosis reveals a therapeutic effect of palmitoylethanolamide. Eur. J. Neurosci.

[42] Ribes S (2010). Toll-like receptor stimulation enhances phagocytosis and intracellular killing of nonencapsulated and encapsulated Streptococcus pneumoniae by murine microglia. Infect. Immun..

[43] Nau R, Ribes S, Djukic M, Eiffert H (2014). Strategies to increase the activity of microglia as efficient protectors of the brain against infections. Front. Cell. Neurosci.

[44] Redlich S, Ribes S, Schutze S, Nau R (2014). Palmitoylethanolamide stimulates phagocytosis of *Escherichia coli* K1 by macrophages and increases the resistance of mice against infections. J. Neuroinflammation.

[45] Masek K, Perlík F, Klíma J, Kahlich R (1974). Prophylactic efficacy of N-2-hydroxyethyl palmitamide (impulsin) in acute respiratory tract infections. Eur. J. Clin. Pharmacol..

[46] Redlich S, Ribes S, Scutze S, Czesnik D, Nau R (2012). Palmitoylethanolamide stimulates phagocytosis of Escherichia coli K1 and Streptococcus pneumoniae R6 by microglial cells. J. Neuroimmunol..

[47] Singhrao, S. K., Harding, A., Poole, S., Kesavalu, L. & Crean, S. Porphyromonas gingivalis Periodontal Infection and Its Putative Links with Alzheimer’s Disease. *Mediators Infiamm**.* 137357 (2015). 10.1155/2015/137357 PMC4430664 26063967

[48] Petrosino, S. & Di Marzo, V. The pharmacology of palmitoylethanolamide and first data on the therapeutic efficacy of some of its new formulations. *Br J Pharmacol*. 13580 (2016). 10.1111/bph.13580 PMC5429331 27539936

[49] Kahlich R (1979). Studies on prophylactic efficacy of N-2-hydroxyethyl palmitamide (Impulsin) in acute respiratory infections. Serologically controlled field trials. J. Hyg. Epidemiol. Microbiol. Immunol..

[50] Inoue K (2002). Microglial activation by purines and pyrimidines. Glia.

[51] Miller AM, Stella N (2008). CB2 receptor-mediated migration of immune cells: it can go either way. BR. J. Pharmacol.

[52] Thelen M (2001). Dancing to the tune of chemokines. Nat. Immunol..

[53] Fung S, Cherry AE, Xu C, Stella N (2015). Alkylindole-sensitive receptors modulate microglial cell migration and proliferation. Glia.

[54] Franklin A, Parmentier-Batteur S, Walter L, Greenberg DA, Stella N (2003). Palmitoylethanolamide increases after focal cerebral ischemia and potentiates microglial cell motility. J Neurosci..

[55] Walter L (2003). Nonpsychotropic cannabinoid receptors regulate microglial cell migration. J. Neurosci..

[56] Sacerdote P (2005). The nonpsychoactive component of marijuana cannabidiol modulates chemotaxis and IL-10 and IL-12 production of murine macrophages both *in vivo* and *in vitro*. J. Neuroimmunol..

[57] Gonsiorek W (2000). Endocannabinoid 2-arachidonyl glycerol is a full agonist through human type 2 cannabinoid receptor: antagonism by anandamide. Mol Pharmacol..

[58] Sugiura T (2000). Evidence that 2-arachidonoylglycerol but not N-palmitoylethanolamine or anandamide is the physiological ligand for the cannabinoid CB2 receptor. Comparison of the agonistic activities of various cannabinoid receptor ligands in HL-60 cells. J Biol Chem.

[59] Martin-Moreno AM (2011). Cannabidiol and other cannabinoids reduce microglial activation *in vitro* and *in vivo*: relevance to Alzheimer’s disease. Mol. Pharmacol..

[60] Di Marzo V (2001). Palmitoylethanolamide inhibits the expression of fatty acid amide hydrolase and enhances the anti-proliferative effect of anandamide in human breast cancer cells. Biochem. J..

[61] Clark A, Wodarski R, Guida F, Sasso O, Malcangio M (2010). Cathepsin S release from primary cultured microglia is regulated by the P2X7 receptor. Glia.

[62] Luongo L (2014). The A1 adenosine receptor as a new player in microglia physiology. Glia.

[63] Iannotti FA, Barrese V, Formisano L, Miceli F, Taglialatela M (2013). Specification of skeletal muscle differentiation by repressor element-1 silencing transcription factor (REST)-regulated Kv7.4 potassium channels. Mol. Biol. Cell..

[64] Matias I, Gonthier MP, Petrosino S, Docimo L, Capasso R, Hoareau L, Monteleone P, Roche R, Izzo AA, Di Marzo V (2007). Role and regulation of acylethanolamides in energy balance: focus on adipocytes and beta-cells. Br J Pharmacol.

[65] Piscitelli F, Carta G, Bisogno T, Murru E, Cordeddu L, Berge K, Tandy S, Cohn JS, Griinari M, Banni S, Di Marzo V (2011). Effect of dietary krill oil supplementation on the endocannabinoidome of metabolically relevant tissues from high-fat-fed mice. Nutr Metab.

