# Supplementary material for: Palmitoylethanolamide induces microglia changes associated with increased migration and phagocytic activity: involvement of the CB2 receptor
Source: Sci Rep. 2017 Mar 23;7:375. doi: 10.1038/s41598-017-00342-1 (PMC5428303; doi:10.1038/s41598-017-00342-1)
Supplement: Supplementary file 1 — Supplementary Information [file 41598_2017_342_MOESM1_ESM.doc]

**Palmitoylethanolamide induces microglia changes associated with increased migration and phagocytic activity: involvement of the CB2 receptor**

F. Guida^1,5#^, L. Luongo^1,5#^, S. Boccella^1^, M.E. Giordano^1^, R. Romano^1^, G. Bellini^2^, I. Manzo^2^, A. Furiano^1^, A. Rizzo^3^, R. Imperatore^4,5^, F.A. Iannotti^4,5^, E. D’Aniello^4,5^, F. Piscitelli^4,5^, F.sca Rossi^2^, L. Cristino^4,5^, V. Di Marzo^4,5^, V de Novellis^1,5^ and S. Maione^1,5*^

^1^Department of Experimental Medicine, Section of Pharmacology L. Donatelli, Università degli Studi della Campania “Luigi Vanvitelli” (Ex SUN), 80138 Naples, Italy.

^2^Department of Women, Child and General and Specialistic Surgery, Università degli Studi della Campania “Luigi Vanvitelli” (Ex SUN) 80138 Naples, Italy.

^3^Department of Experimental Medicine, Section of Microbiology and Clinical Microbiology, Università degli Studi della Campania “Luigi Vanvitelli” (Ex SUN), 80138, Naples, Italy.

^4^Institute of Biomolecular Chemistry, Consiglio Nazionale delle Ricerche, Pozzuoli, Italy

^5^ Endocannabinoid Research Group, Institute of Biomolecular Chemistry, C.N.R., Pozzuoli Italy

**Supplemental Experimental Procedures**

***Cell culture***

*-Microglia.* Primary microglia were prepared from SD postnatal rats (P2-3). Each culture was made from two or three cortex. Tissues were isolated, dissociated in trypsin solution and suspended in Dulbecco’s modified Eagle medium (DMEM) with 15% heat-inactivated fetal bovine serum (GIBCO) and 1% penicillin/streptomycin (Sigma). The cell suspension was filtered through a 100-µm nylon mesh and plated in tissue culture flasks precoated with poly-D-lysine (10 mg/mL). Cells were maintained in a 5% CO_2_ incubator at 37°C and were harvested as floating cell suspensions following shaking after 10–12 days (Clark et al., 2010; Luongo et al., 2014). Cells were plated in 6 multiwells for mRNA and protein extraction or in 8-chamber slides for immunocytochemistry staining. Cell viability was determined by measuring the lactate dehydrogenase (LDH assay kit, Sigma, UK) according to the manufacturers’ instructions. In all experiments no significant difference was observed between not treated and vehicle-treated cells (DMSO 0.01%).

*-Macrophages.* Eight healthy volunteers were enrolled as whole blood donors for macrophages cultures after written informed consent with the approval of the Ethic Committee of the Second University of Naples and in compliance with the national legislation and the Declaration of Helsinki. Ficoll gradient centrifugation (Hystopaque 1077 density gradient; Sigma-Aldrich, St Louis, MO, USA) was performed to isolate peripheral blood mononuclear cells (PBMCs) from whole blood. PBMCs were then diluted at 10^6^ cells/ml in α-Minimal Essential Medium (α-MEM; Lonza, Verviers, Belgium) supplemented with 10% Fetal Bovine Serum (FBS; Lonza, Verviers, Belgium), 100 IU/ml penicillin, 100 g/ml streptomycin (Gibco Limited, Uxbridge, UK), and 2mM L-glutamine (Lonza, Verviers, Belgium), and harvested for 14 days in presence of 25 ng/ml recombinant human macrophage colony-stimulating factor (rh-MCSF; Prepotech, London, UK) in order to obtain macrophages cultures. Human macrophages were differentiated in 6- or 12-multiwell for mRNA and protein extraction, respectively, or in 8-chamber slides for immunocytochemistry staining.

*-Treatments*

Cells were incubated with ultra micronized (0.8-6.0 µm) Palmitoylethanolamide, or PPAR-α agonist GW7647 alone or with PPAR-α selective antagonist GW6471, CB2 selective inhibitor AM630 or CB1 selective inhibitor AM251.

***Real Time Polymerase Chain Reaction***

To quantify the expression levels of CB2 mRNA, three serial complementary DNA (cDNA) dilutions (1:5) obtained from reverse transcription of 100 ng total mRNA (High Capacity cDNA Reverse Transcription kit; Applied Biosystems, Foster City, CA, USA) were amplified by Real Time polymerase chain reaction (Real Time PCR or qPCR) with SYBR green as fluorophore. The housekeeping gene β-actin was used as endogenous control. Assays were performed at least in triplicate. The 20 µl reaction mixture contained 4 μl [20 ng/μl] cDNA, 10 μl Fast SYBR Green Master Mix (Applied Biosystems, Foster City, CA, USA), 3.6 μl [20 µM] primers mix, and sterile water. The thermal cycling program consisted of 10 min at 95°C, followed by 40 cycles at 95°C for 15 sec and 60°C for 1 min (7900 HT Fast System Thermal Cycler; Applied Biosystems, Foster City, CA, USA). Gene expression profiling was achieved using the comparative cycle threshold method of relative quantification to β-actin. Real Time-PCR products were analyzed using the SDS software (Applied Biosystems, Foster City, CA, USA). The experiment has been performed by a blind observer at least three times.

***Phagocytosis assay***

Microglial cells were seeded in 24-well plates at a density of 35000 cells/ml and were pretreated with PEA (100 nM), and/or GW6471 (10 µM), and/or AM630 (100 nM) for 24 hours and incubated in 5% CO_2_ at 37°C before infection. The *P. gingivalis* cells (MOI=100), approximately in the late logarithmic growth phase, washed and resuspended in PBS, were added and incubated in 5% CO_2_ at 37°C for another 90 min. After incubation, each well was washed twice with PBS and incubated with Dulbecco’s modified Eagle’s medium (DMEM, Gibco Invitrogen, Milan, Italy) containing 100 μg/ml gentamicin for 60 min at 37°C in 5% CO_2_ to kill extracellular bacteria. After incubation with the antibiotic, microglial cells were washed twice with PBS and lysed in cold distilled water. Ingested bacteria were serially diluted and spread on plates for viable counts. The CFU of *P. gingivalis* were counted after suitable dilutions of the lysates were plated on TS agar and incubated for 24 hours at 37°C. All serial dilutions were in cell-culture medium. The number of *P. gingivalis* was determined and expressed as percentage. Bacteria were tested in three separate assays; each assay was the average of the triplicate wells. Bacterial strain *P. gingivalis* from the American Type Culture Collection (ATCC 33277) was grown anaerobically at 37°C for 2–days in Trypticase soy (TS) broth (30 g/l) containing 1 g/l yeast extract (Difco), 1 g/l glucose, 0.5 g/l potassium nitrate, 1 ml/l sodium lactate (Sigma L-1375), 0.5 g/l sodium succinate and 1 g/l sodium fumerate; after autoclaving, filter-sterilized supplements were added (0.4 g/l sodium carbonate; 0.005 g/l hemin [r H-2250]; 0.4 g/l cysteine; and 0.001 g/l vitamin K [r M-5625]). Late logarithmic-phase cells were employed; bacteria were washed twice in sterile PBS.

***Intracellular survival assay***

To monitor survival inside treated microglial cells for 24 hours were incubated with *P. gingivalis* (MOI=100) for 90 min, at a bacteria/cell ratio of 100:1.Thereafter, cells were washed twice with PBS and to destroy extracellular bacteria, incubated in culture medium containing gentamicin (100 μg/ml). After 4 hours, the monolayers were washed with PBS and lysed with cold distilled water. The amounts of intracellular bacteria were determined as CFU by quantitative plating of serial dilutions on TS agar, and incubated for 24 hours at 37°C. All serial dilutions were in cell-culture medium. The number of *P. gingivalis*  was determined and expressed as percentage. Bacteria were tested in three separate assays; each assay was the average of the triplicate wells.

***Migration assay***

Migrating microglial cells were analyzed in a modified chemiotaxis plate (iuvo Chemotaxis Assay plate, Thermo Scientific Inc., Germany). One hundred microliters of 1–2.5×10^5^ microglial cells per cm^2^ were seeded in 5 mm cell port 24 hours before the migration assay in presence or absence of PEA (100 nM) or PPAR-α agonist GW7647 (1 µM). Twenty microliters of assay medium containing either PPAR-α selective antagonist GW6471 (10 µM) or CB2 selective inhibitor AM630 (100 nM) or CB1 selective inhibitor AM251 (500 nM) or vehicle were added 15 min before the addition of PEA or GW7647. PEA, GW7647, GW6471 and AM630 were dissolved in PBS containing DMSO (final concentration 0.01%). Microphotographs of migrating microglial cells were monitored with inverted microscopy system (Leica DMI6000) with differential interference contrast transmission filter. 30 min after 20 µL of 2-AG (diluted stock in ethanol) were added to the attractant port permeating through the unit and creating a chemo-attractant gradient in the gradient channel. The concentration of 2-AG (100 µM) used for the migration assay derived by previous experiments in which three different concentrations have been used. A source of ATP (500 µM) (PeproTech, UK) was used to verify migratory capability of the cells. The movement of microglia was recorded each 5 min for 90 min starting from the application of 2-AG. Using DAPI image-based detection the number of migrating cells was determined counting the migrated cells from the plates where microglial cells, treated with PEA or GW7647 in presence or absence of GW6471 or AM630 or AM251, were exposed to vehicle or 2-AG chemo-attractant ligand. Non-treated cultured microglial cells were maintained in incubation media during the relative treatment time with or without vehicle (DMSO 0.01%). No significant difference was observed between not treated and vehicle-treated cells.

**Supplementary figures**

**S1**

**
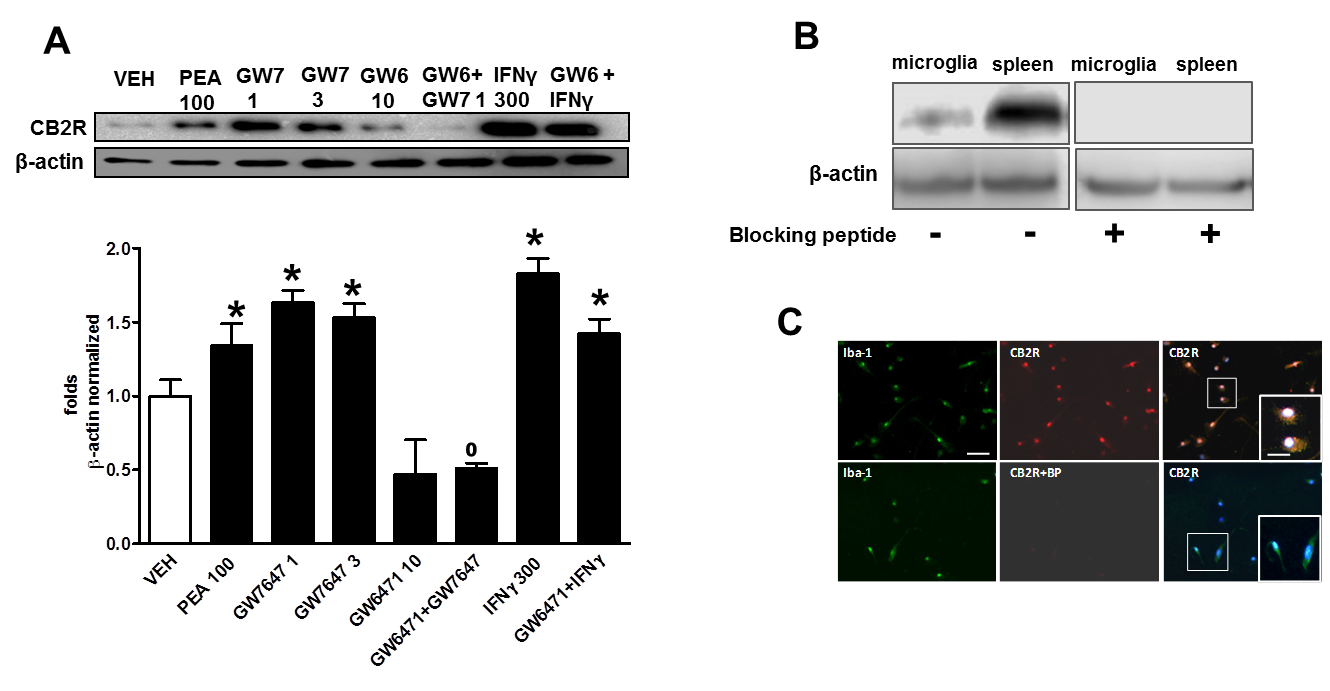
**

S1. **PEA induces CB2R expression in microglia.** A: Representative western blot image and related quantification showing the expression of CB2R in microglial cell lysates in presence of 24 hours of treatment with PEA (100 nM), GW7647 (1 µM), GW7647 (3 µM), GW6471 (10 µM), GW6471 + GW7647, IFNγ (300U/ml), GW6471 + IFNγ, using the housekeeping β-actin as loading control. Data are shown as mean ± SD (n = 6-8)**.** **p*<0.05 compared to vehicle-treated cells, ⁰ *p*<0.05 compared to GW7647 (1 µM)-treated cells. One way ANOVA, post-hoc Tukey’s. B: Representative western blot image of CB2R in microglia cells or spleen and after CB2R targeted blocking peptide incubation. β-actin was used as loading control. C: CB2R expression (red) in Iba-1 labeled microglia (green) (upper panel), and after CB2R targeted blocking peptide incubation (lower panel). Notice the absence of CB2R staining in the overexposed picture. Scale bar = 100 µm and 25 µm for cropped insert.

**S2**

**
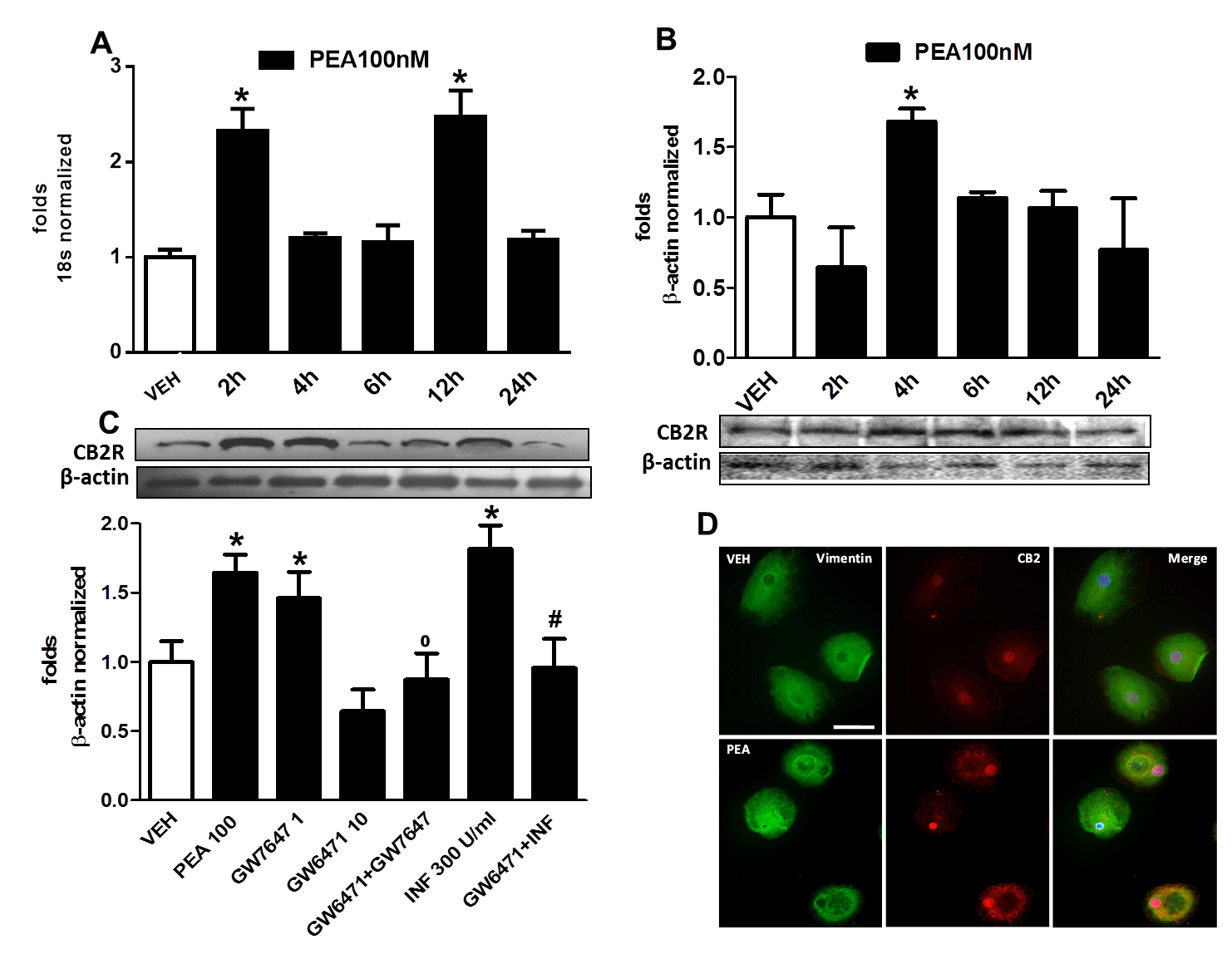
**

**S2. PEA induces CB2R expression in human macrophages cell cultures.** A: Real-time PCR analyses of CB2 m-RNA in cultured macrophages following PEA incubation (100 nM for 2, 4, 6, 12 or 24 hours), using 18S as loading control. **p*<0.05 compared to vehicle-treated cells. One way ANOVA, post-hoc Tukey’s. B: Representative western blot image and quantification showing the expression of CB2R in macrophage cell lysates following incubation with PEA (100 nM for 2, 4, 6, 12 or 24 hours). C: Representative western blot image and quantification showing the expression of CB2R in macrophage cell lysates following 4 hours of incubation with PEA 100 nM, GW76471 µM, GW6471 10 µM, GW6471 + GW7647, INFγ 300U/ml, GW6471 + INFγ, using β-actin as loading control. Data are shown as mean ± SD (n = 6-8)**.** **p*<0.05 compared to vehicle, ⁰ *p*<0.05 compared to GW7647-treated cells, # *p*<0.05 compared to IFNγ-treated cells. One way ANOVA, post-hoc Tukey’s. D: CB2R staining (red) in macrophages, identified as vimentin labeled cells (green) following PEA incubation. Scale bar = 25 μm.

**S3**

**
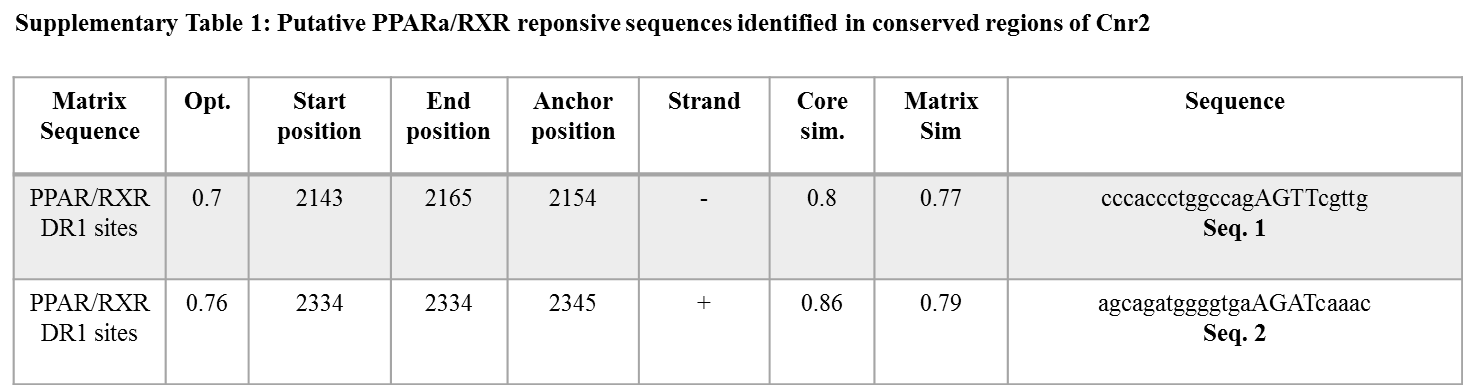
**

S3. Putative PPARa/RXR responsive sequences identified in conserved regions of Cnr2

**S4**


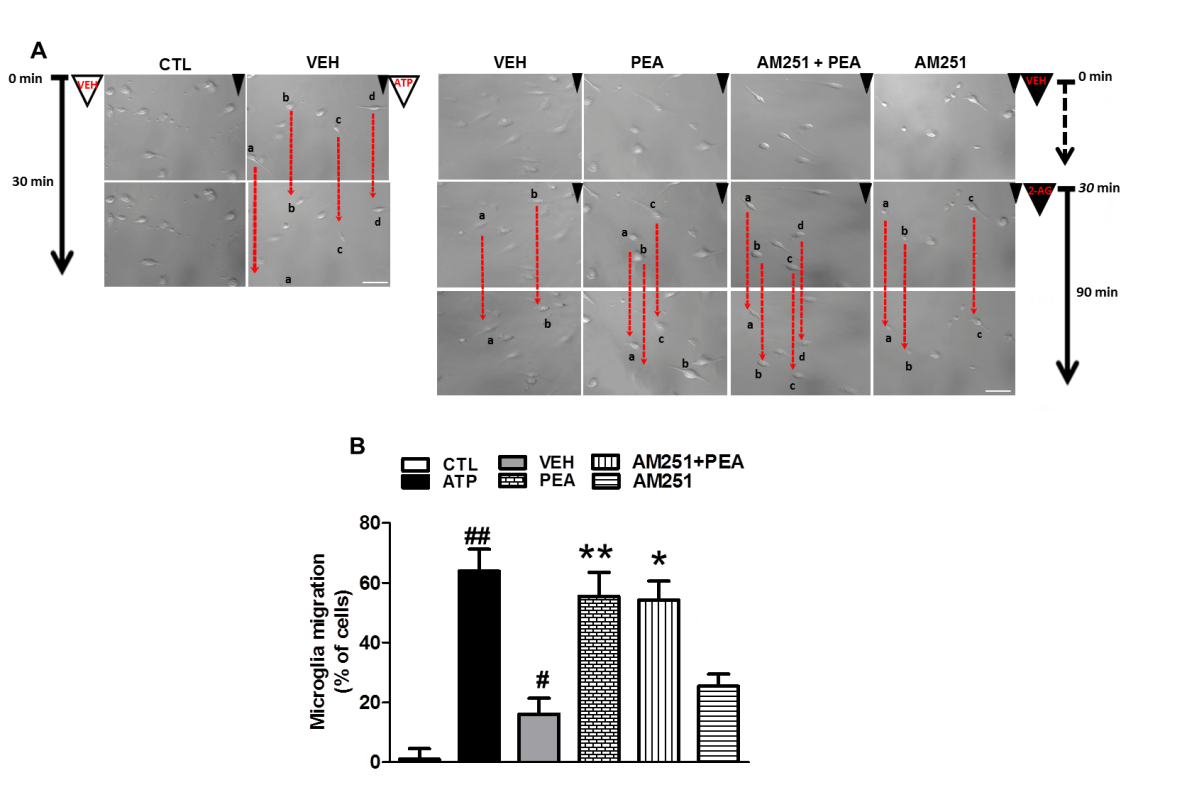


S4. **PEA does not induce microglia migration towards a 2-AG source through CB1R**. (A): Image-based detection of 2-AG (100 µM) chemoattractive effect on cultured microglial cells after different treatments. Panel shows the migratory effect induced by PEA incubation (100 nM), alone or in the presence of AM251 (500 nM) or AM251 alone as compared to the control group (vehicle). Migratory effect induced by ATP (500 µM) was used as positive control of cells motility. Representative data of microglial cell time-lapse migration recorded at starting point (0 min), 30 and 90 min from the 2-AG or 30 min from ATP exposure. (B): The quantification indicates the percentage of microglia cells affected by 2-AG or ATP chemoattractive movements vs total cell number at 30 or 90 min. Data are shown as mean ± S.E.M. (n = 6-8)**.** # P<0.05 and ## P<0.001 compared to CTL; *P<0.05 and **P<0.001 compared to vehicle. One way ANOVA, post-hoc Tukey’s. Scale bar = 50 µm.

**S5**

**S5. Quantification of eCBs and related mediators in microglial cells**. Levels of endogenous 2-arachidonoylglycerol (2-AG), anandamide (AEA) and palmitoylethanolamide (PEA) in microglial cells treated with vehicle (VEH), VEH + *P. gingivalis* or PEA + *P. gingivalis*. Data are shown as means ± SD of separate determinations in N = 3 distinct group of cells. ^*^P < 0.05 vs VEH and VEH+*P. gingivalis* by one way ANOVA, followed by post-hoc Tukey’s test. Note that endogenous PEA levels in microglia incubated with PEA are not shown as they are obviously biased by the amounts of exogenous PEA.

**S6**

**A**


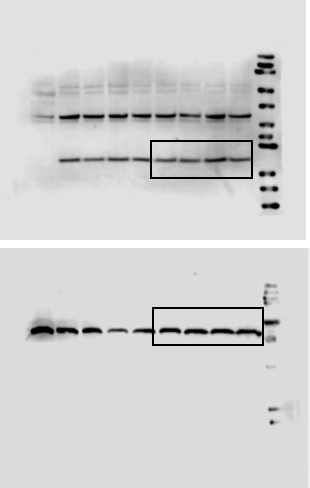


**B**

**S6. Full-length blots/gels of Figure 1B.** A: Representative western blot image of CB2R bands in microglia cells. The four bands of approximately 40 kDa size shown in the box indicate the relative protein expression in different groups of treatment represented in the main figure 1B. B: Representative western blot image of β-actin in the same samples.

**S7**

**A**

**B**


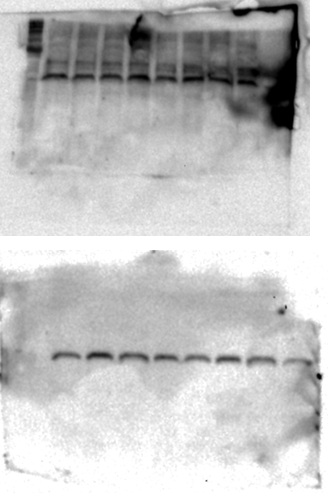


**S7. Full-length blots/gels of Figure 1E.** A: Representative western blot image of CB2R bands in microglia cells. The bands of approximately 40 kDa size indicate the relative protein expression in different groups of treatment represented in the main figure 1E. B: Representative western blot image of β-actin in the same samples.
